# Cognition in Recent Suicide Attempts: Altered Executive Function

**DOI:** 10.3389/fpsyt.2021.701140

**Published:** 2021-07-22

**Authors:** Jessica Fernández-Sevillano, Susana Alberich, Iñaki Zorrilla, Itxaso González-Ortega, María Purificación López, Víctor Pérez, Eduard Vieta, Ana González-Pinto, Pilar Saíz

**Affiliations:** ^1^Department of Neuroscience, Universidad del País Vasco/Euskal Herriko Unibertsitatea, Bilbao, Spain; ^2^Department of Psychiatry, Bioaraba Research Institute, Araba University Hospital, Vitoria-Gasteiz, Spain; ^3^Biomedical Research Networking Centre in Mental Health (CIBERSAM), Madrid, Spain; ^4^Institut Hospital del Mar d'Investigacions Mèdiques (IMIM) Department of Psychiatry, Institut de Neuropsiquiatria i Addicions, Hospital del Mar, Universidad Autónoma de Barcelona, Barcelona, Spain; ^5^Hospital Clinic, Institute of Neuroscience, University of Barcelona, Institut D'Investigacions Biomèdiques August Pi i Sunyer (IDIBAPS), Barcelona, Spain; ^6^Department of Psychiatry, Universidad de Oviedo, Instituto de Investigación Sanitaria del Principado de Asturias, Servicio de Salud del Principado de Asturias, Oviedo, Spain

**Keywords:** suicide, major depressive disorders, cognition, executive function, trauma

## Abstract

**Background:** Neuropsychological alterations can lead to inaccurate perception, interpretation, and response to environmental information, which could be a risk factor for suicide.

**Methods:** Ninety-six subjects were recruited from the Psychiatry Department of the Araba University Hospital—Santiago, including 20 patients with a recent attempt and diagnosis of major depressive disorder (MDD) according to DSM-V, 33 MDD patients with history of attempted suicide, 23 non-attempter MDD patients, and 20 healthy controls. All participants underwent a clinical interview and neuropsychological assessment on the following cognitive domains: working memory, processing speed, decision-making, executive function, and attention. Backward multiple regressions were performed adjusting for significant confounding variables. For group comparisons, ANOVA and Bonferroni *post-hoc* tests were performed with a *p* < 0.05 significance level.

**Results:** The patient groups did not differ regarding severity of depression and stressful events in the last 6 months. In comparison to healthy controls, depressed patients with lifetime suicide attempts had more general trauma (*p* = 0.003), emotional abuse (*p* = 0.003), emotional negligence (*p* = 0.006), and physical negligence (*p* = 0.009), and depressed patients with recent suicide attempts had experienced more child sexual abuse (*p* = 0.038). Regarding neuropsychological assessment, all patient groups performed significantly worse than did healthy controls in processing speed, decision-making, and attention. Comparisons between patient groups indicated that recent suicide attempters had poorer performance on executive function in comparison to both depressed lifetime attempters and depressed non-attempters (*B* = 0.296, *p* = 0.019, and *B* = 0.301, *p* = 0.028, respectively). Besides, women with recent attempts had slightly better scores on executive function than males. Regarding the rest of the cognitive domains, there were no significant differences between groups.

**Conclusion:** Executive function performance is altered in recent suicide attempts. As impaired executive function can be risk factor for suicide, preventive interventions on suicide should focus on its assessment and rehabilitation.

## Introduction

Suicide is a major health issue worldwide involving nearly 800,000 deaths per year (1). Identifying risk factors can lead to a better understanding and prevention of this behavior (2). Age, sex, history of previous attempts, genetic predisposition, drug consumption, and early adversity, among others, have been extensively reported as relevant features that can predict a suicide attempt (3–6).

Additionally, neuropsychological performance has been linked to suicide in several studies across all the life span. Impaired complex cognition, social cognition, and episodic memory (7, 8) were reported in adolescents at risk of suicide. In children with a family history of suicide attempts, cognitive, attention, and language reasoning have been described, increasing their risk of reproducing a suicide attempt themselves (9). In contrast, McHugh et al. (10) found better cognitive functioning on processing speed, verbal learning, working memory, delayed memory, and verbal fluency in suicidal youngsters and young adults.

In adult and elderly populations, many studies have provided evidence of alterations in executive function (11–13), attention (14), memory (15), and decision-making (13, 16, 17) in suicide attempters. Deficits in these cognitive domains, especially executive function that organizes and directs behavior, can lead to an inaccurate perception, interpretation, retrieval, and response to environmental information, which may result in an inflexible, pessimistic way of thinking about their future and themselves (18). Thus, these cognitive deficits may imply a higher risk of suicide as a response to adverse life events. In summary, existing evidence supports a connection between cognition and suicide. However, these studies do not determine the specific cognitive alterations involved in a recent suicide attempt, that is, the current altered processes that surround and may contribute to suicidal behavior. Besides, these cognitive alterations are not unique to suicide and have also been described in depressive patients (19–24); therefore, it remains unclear which specific cognitive domains are altered in recent suicidal behavior. Considering data scarcity, the aim of this study was to explore cognitive alterations in recent suicide attempts. For this purpose, we compared the neuropsychological performance of depressed patients with a recent attempt, depressed patients with a history of suicide, depressed non-attempters, and healthy controls on the following cognitive domains: working memory, processing speed, decision-making, executive function, and attention.

## Materials and Methods

### Participants

Ninety-six participants were recruited from the Psychiatry Department of the Araba University Hospital—Santiago. All patients were diagnosed of major depressive disorder (MDD) according to the Diagnostic and Statistical Manual of Mental Disorders, 5th edition (DSM-5) criteria and were receiving psychopharmacological treatment for their condition. The sample was categorized into the following groups: 20 depressed patients who were hospitalized after a recent suicide attempt (≤ 30 days), 33 patients with a past suicide attempt during their lifetime and hospitalized for MDD episodes, 23 without history of suicidal attempts and hospitalized for MDD episodes, and 20 healthy controls with no personal or family history of mental illness matched by age and sex with a reference group, that is, a recent suicide attempt group. Suicide attempt was defined as a “self-initiated sequence of behaviors by an individual who, at the time of the initiation, expected that the set of actions would lead to his or her own death” (DSM-5). The exclusion criteria for the three groups including patients were: presence of psychotic symptoms and other comorbid psychiatric disorders with the exception of tobacco use disorder, acute infections, neurological illness, intellectual disability, dementia, organic diseases that compromise cognitive functioning, and cognitive syndromes. In addition to this, the exclusion criteria for healthy controls included either personal or a family history of major psychiatric disorders. All participants were between 18 and 65 years old and had signed an informed consent. This study was approved by the ethical board of the Araba University Hospital and was conducted according to the Declaration of Helsinki (25).

### Procedure and Measures

After recruitment and signature of the informed consent, each participant had an interview with a psychologist for sociodemographic data collection and clinical assessment of depressive symptoms, stressful events, and childhood trauma. Depressive symptom severity was assessed using the 17-item Spanish version of the Hamilton Depression Rating Scale (HDRS) (26, 27), a structured instrument widely used in the psychiatric field (28–30) that offers a quantitative measure of the severity of depressive symptoms in a clinical population according to the criteria of the evaluator who conducts the clinical interview. Each item has between three and five possible responses with 0–2 or 0–4 scores, respectively, and the total score ranges from 0 to 52. The 17-item Spanish version has good reliability, with a Cronbach's α of 0.72, and validity with a correlation with other scales for depressive symptoms (Montgomery–Asberg and Beck's Depression Inventory) ranging from 0.8 to 0.9. Recent (6 months) stressful events were measured using the Spanish version of the List of Threatening Experiences (LTE) (31, 32), a 12-item brief questionnaire with yes/no responses regarding personal, relational, financial, and health problems. This questionnaire has a high test–retest reliability (κ = 0.61–0.87) and is a valid and reliable measure of stressful events in mental health, specifically in depression (OR = 1.64–2.57) (32), which has been previously used in studies with psychiatric populations (33–36). Childhood abuse and neglect were assessed with the Spanish version of the Childhood Trauma Questionnaire—Short Form (CTQ-SF) (37), a self-administered 28-item questionnaire with five Likert responses (never, rarely, sometimes, often, and always) that has been extensively used (38–43) to evaluate the maltreatment dimensions that were detected in the original factorial analysis for construct validity (44): emotional (Cronbach's α = 0.87), physical (Cronbach's α = 0.89) and sexual abuse (Cronbach's α = 0.94) and physical (Cronbach's α = 0.66) and emotional (Cronbach's α = 0.83) neglect (37).

Besides, the participants underwent a neuropsychological assessment using a standardized test on the following cognitive domains: working memory, processing speed, decision-making, executive function, and attention. The test and measures used in each domain (Table 1) were adapted from previous existing literature on cognitive assessment in mental disorders (50, 51).

**Table 1:** Cognitive domains.

Working memory	WAIS-IV Arithmetic: total score WAIS-IV Digit: total score Wechsler (45)
Processing speed	WAIS-IV Symbol Search: total score WAIS-IV Coding: total score Wechsler (45)
Decision-making	Iowa gambling test: total money score Bechara et al. (46)
Executive function	Wisconsin sorting card test: categories, errors, perseverative errors Heaton (47) Stroop test: interference score Golden (48) FAS test: total correct answers Buriel et al. (49)
Attention	Stroop test: color word Golden (48)

### Statistical Analyses

Statistical analysis was performed by an expert biomedical statistician. Data were checked for Gaussian distribution. Comparisons between groups regarding the sociodemographic and clinical variables were performed with ANOVA followed by Bonferroni *post-hoc* testing for continuous variables and chi-square for categorical variables. For the analyses of cognitive performance, measures selected from each test (Table 1) were gathered into the corresponding cognitive domain that is evaluated and the obtained scores were transformed to average *z*-scores. Backward multiple regressions were performed adjusting for the following confounding variables: age, sex, economic status, education, marital status, drug consumption (tobacco, cannabis, and alcohol), and severity of depressive symptoms according to the HDRS. In each model, only significant confounders were taken into account.

## Results

### Sociodemographic and Clinical Variables

There were no significant differences between groups regarding sociodemographic variables (Table 2). In the recent attempt group, 35% of patients were recruited after an index attempt, 40% of patients reattempted for the second time, and 60% of patients had more than two reattempts. In the lifetime attempt group, 6.3% of patients had attempted suicide once, 34.4% twice, and 59.3% more than twice. Regarding psychiatric medication, the majority had similar treatments as 94% were under polytherapy consisting of antidepressants and benzodiazepines, whereas 6% of patients were under monotherapy of either of those treatments.

**Table 2:** Demographic and clinical variables.

		**Recent** **attempters** **(*n* = 20)**	**Lifetime** **attempters** **(*n* = 33)**	**Depressed non-attempters** **(*n* = 23)**	**Healthy** **controls** **(*n* = 20)**	**Statistical** **contrast** **(*p*)**
Sex, *n* (%)	Female	13 (65%)	26 (78.8%)	18 (78.3%)	14 (70%)	*X*^2^ = 1.617; *p* = 0.656
	Male	7 (35%)	7 (21.2%)	5 (21.7%)	6 (30%)	
Age, mean (SD)		44.70 (8.785)	44.45 (12.765)	50.57 (9.926)	44.58 (9.221)	*F*_(3, 91)_ = 1.842; *p* = 0.145
Education level, *n* (%)	Primary	2 (10%)	6 (18.2%)	5 (21.7%)	0 (0%)	*X*^2^ = 9.886; *p* = 0.360
	Secondary	7 (35%)	11 (33.3%)	7 (30.4%)	6 (30%)	
	Preparatory	5 (25%)	9 (27.3%)	6 (26.1%)	4 (20%)	
	University	6 (30%)	7 (21.2%)	5 (21.7%)	10 (50%)	
Economic status, *n* (%)	Low	7 (38.9%)	10 (37%)	10 (50%)	6 (33.3%)	*X*^2^ = 5.611; *p* = 0.468
	Medium	9 (50%)	11 (40.7%)	10 (50%)	9 (50%)	
	High	2 (11.1%)	6 (22.3%)	0	3 (16.7%)	
Previous suicide attempts, mean (SD)		1.47 (1.87)	2.34 (2.04)	–	–	*t* = −1.52; *p* = 0.1
Age of index attempt, mean (SD)		33.55 (11.91)	31.83 (15.35)	–	–	*t* = 0.42; *p* = 0.67
Previous depressive episodes, mean (SD)		2.60 (2.90)	7.50 (9.81)	2.38 (1.59)	–	*F*_(2, 47)_ = 2.16; *p* > 0.05
Depression severity (HDRS), mean (SD)		15.50 (7.23)	17.87 (6.54)	14.36 (8.93)	1.60 (2.09)	*F*_(3, 89)_ = 25.741; *p* < 0.01
Stressful events (LTE), mean (SD)		2.80 (1.70)	2.53 (2.11)	2.56 (1.90)	0.5 (1.15)	*F*_(3, 91)_ = 7,281; *p* < 0.01
CTQ total, mean (SD)		57 (25.63)	64.1 (21.66)	52.57 (23.06)	38.15 (7.12)	*F*_(3, 80)_ = 4.54; *p* < 0.05
CTQ emotional abuse, mean (SD)		11.15 (6.63)	13.19 (6.16)	10.38 (6.08)	7.57 (2.90)	*F*_(3, 82)_ = 3.112; *p* < 0.05
CTQ physical abuse, mean (SD)		8.80 (4.93)	8.13 (5.02)	8.10 (5.73)	6.21 (2.36)	*F*_(3, 82)_ = 0.813; *p* = 0.49
CTQ sexual abuse, mean (SD)		10 (7.48)	8.81 (5.58)	6.14 (2.15)	5.07 (0.27)	*F*_(3, 82)_ = 3.789; *p* < 0.05
CTQ emotional negligence, mean (SD)		10.2 (5.02)	14.13 (6.07)	10.67 (5.94)	8.08 (1.85)	*F*_(3, 80)_ = 4.736; *p* < 0.05
CTQ physical negligence, mean (SD)		11.55 (2.58)	13.06 (3.18)	11.68 (3.48)	9.93 (1.90)	*F*_(3, 83)_ = 3.770; *p* < 0.05

*HDRS, Hamilton Depression Rating Scale; LTE, List of Threatening Experiences; CTQ, Childhood Trauma Questionnaire*.

In addition to having similar medication profiles, the severity of depressive symptoms was equally distributed across groups. Bonferroni *post-hoc* analyses revealed that all groups had significantly higher scores for depressive symptoms measured by the HDRS than did the healthy controls (*p* < 0.01 in each comparison), but the severity of depression was similar across patient groups. Besides, healthy controls had fewer stressful events in the last 6 months than each of the patient groups (*p* < 0.05 in each comparison).

Also, *post-hoc* analyses of the CTQ-SF scores indicate greater general trauma (*p* = 0.003), emotional abuse (*p* = 0.003), emotional negligence (*p* = 0.006), and physical negligence (*p* = 0.009) in patients with a history of suicide attempts in comparison to healthy controls. Besides, patients with recent suicide attempts reported higher scores of child sexual abuse (*p* = 0.038) than did healthy controls.

### Performance on Cognition

The scores on the five domains included in this study are presented in Table 3.

**Table 3:** Cognitive domains by group^a^.

	**Recent attempters** **(*n* = 20)**	**Lifetime attempters** **(*n* = 33)**	**Depressed non-attempters** **(*n* = 23)**	**Healthy controls** **(*n* = 20)**
Working memory	−0.577 (0.822)	−0.825 (0.867)	−0.798 (0.845)	0 (0.784)
Processing speed	−1.002 (0.821)	−1.403 (1.444)	−1.306 (1.411)	0 (0.944)
Decision making	−0.772 (1.089)	−0.726 (1.053)	−0.873 (0.889)	0 (1.000)
Executive function	−0.191 (0.256)	0.066 (0.484)	0.070 (0.530)	0.021 (0.313)
Attention	−0.910 (0.770)	−1.025 (0.667)	−0.913 (0.646)	0.046 (0.781)

a *Data are presented as means and standard deviations. All values are z-scores*.

Backward stepwise multiple regressions were performed and adjusted by significant confounders (age, sex, economic level, marital status, years of education, severity of depression, and substance consumption) with healthy controls as the reference group. Healthy controls performed significantly better in processing speed, decision-making, and attention than did all groups of depressed patients, with no differences between them (Table 4).

**Table 4:** Multiple regression^a^.

	**Working memory**	**Processing speed**	**Decision-making**	**Executive function**	**Attention**
Recent attempters	−0.113	−1.065^*^	−0.829^*^	−0.229	−0.604^*^
Lifetime attempters	−0.221	−1.416^**^	−0.664^*^	0.068	−0.577^*^
Depressed non-attempters	−0.313	−1.050^*^	−0.782^*^	0.072	−0.567^*^

* *p < 0.05;*

** *p < 0.001.*

a *Data are presented as adjusted B coefficients assuming “healthy controls” as the reference group*.

As the main objective of this study was to determine the cognitive differences between recent suicide attempters and current non-suicidal depressed patients, the reference group for adjusted regressions was afterwards changed to recent attempters. The results yielded significant differences on the executive function domain. Both lifetime attempters and depressed non-attempters had significantly higher scores in this domain in comparison to recent attempters (*B* = 0.296, *p* = 0.019, and *B* = 0.301, *p* = 0.028, respectively); that is, recent suicide attempters had poorer performance on executive function. However, there were no significant differences between groups in the rest of the cognitive domains.

Further analyses showed that there was an interaction between group (depressed recent attempters, depressed lifetime attempters, and depressed non-attempters) and sex on executive performance (Table 5). According to these results, women with recent attempts had slightly better scores on executive function than did males. However, in the rest of the groups, men outperformed women, especially in depressed non-attempter patients, with a large effect size indicating a strong interaction between sex and group.

**Table 5:** Effect sizes of being female on executive function^a^.

	**Female^**b**^**
Recent attempters	0.088 (−0.301, 0.478)
Lifetime attempters	−0.239 (−0.624, 0.146)
Depressed non-attempters	−0.663 (−1.134, −0.192)
Healthy controls	−0.189 (−2.058, 1.680)

a *Adjusted for potential confounders.*

b *Effect size is interpreted as the difference in average executive function domain between females and males*.

## Discussion

The main finding of this study is that MDD patients with suicide attempts in the last 30 days have significantly poorer performance on executive function compared to MDD patients with a previous history of suicide attempts and to MDD non-attempter patients. This finding indicates that suicide might be accompanied with an altered performance on executive function. Deficits in this cognitive domain have already been described in patients with a history of suicidality (12, 14, 52–56) and in depressed patients without suicidal behavior (23, 53), especially in those with poor treatment response (57), but to the best of our knowledge, this is the first study that compares MDD patients with recent attempts, MDD patients with lifetime attempts, MDD non-attempter patients, and healthy controls.

Executive function directs thought and behavior toward an objective in order to respond to current or future situational demands. According to the comprehensive model proposed by Miyake et al. (58), this implies the inhibition of responses, shifting between tasks and the constant update of the working memory. Other authors also include planning, decision-making, working memory, and error detection among its functions (59, 60). All in all, these processes allow the deliberate and intentional goal-directed response to environmental stimuli and are subject to the influence of individual factors, such as emotional regulation, personality traits, and even traumatic events (61–63). Deficits in these processes can lead to the dysregulation of emotion, thoughts, and actions (64), which might contribute to considering suicide as a solution under critical circumstances (15). In addition, these alterations may also increase interpersonal difficulties, increasing the risk of attempted suicide (17).

According to our results, sex has an influence on executive function. The slightly better performance of women in the recent attempt group may be possibly explained by the fact that higher impulsivity scores have been related to men (61), which implies a tendency to think and behave with less planning, error detection, cognitive flexibility, and inhibitory control (65), processes that are under control of the executive function. Therefore, men are more likely to make non-adaptive choices under demanding or stressing circumstances as risky behavior or suicide. However, depressed non-attempter men had significantly better executive function performance. This may be due to the fact that women have more severe cognitive symptoms of depression, such as rumination and hypochondria (66), and a greater impact on their functionality (67). Apart from psychological factors, there are also biological differences, such as sex hormones (68, 69), dysregulation of the hypothalamus–pituitary–adrenal (HPA) axis (70), and inflammatory parameters (71), that make depression more adverse for women.

Additionally, the neuropsychological assessment results revealed that all depressed patients with similar depression severity performed significantly worse than did healthy controls in processing speed, decision-making, and attention, but with no difference between groups. This suggests that depressed patients have alterations in these specific dimensions associated with their psychopathological process; that is, major depressive disorder is consistent with previous literature (21, 54, 72, 73). According to our findings, these alterations are different from those associated specifically with recent suicide attempts and, therefore, different cognitive processes are specifically more altered in each condition.

Apart from the neurocognitive deficits, traumatic life events are also associated with a higher risk of suicide. Patients with a history of childhood maltreatment (74–76) and sexual abuse (77) have an increased risk of suicidal behavior in their adult life. In our sample, patients with recent suicide attempts reported more child sexual abuse than the rest of the groups. Patients with a history of suicide reported more general trauma, emotional abuse, and negligence, and in general, patients had more recent stressful events than did the controls. Furthermore, the link between trauma, executive function alterations, and suicidal behavior has also been described by several authors. In a recent study by Zelazny et al. (76), higher scores on executive function were protective against suicide in individuals with childhood maltreatment. Carvalho et al. (78) found that maltreated children performed worse than did non-maltreated children in executive function tests, and according to the study by Dannehl et al. (79), these differences are also present in adults with a history of childhood adversity, especially in those with physical abuse and neglect.

In conclusion, recent suicidal behavior is specifically associated with deficits on executive function, which suggests a cognitive dysfunction on information processing and response, especially in critical events. As executive function can be trained and improved (80), cognitive screening and stimulation may be proposed as a strategy for suicide prevention in at-risk patients. Future studies are needed to identify possible differences between sex in each cognitive domain for a more personalized approach and for a better understanding of early detection vulnerability factors that may enable us to prevent suicide in at-risk patients.

### Limitations

This study has a number of limitations. Recent and past trauma events were assessed using self-administered and self-reported scales in MDD patients who can be biased when reporting this information. As more women have recently attempted suicide than men, the sex-related differences on executive function found in recent attempters must be interpreted with caution, and further confirmatory studies are advisable. Moreover, some factors that have been described in the literature as potentially influencing suicidal behavior in major depression, such as anxiety (81) or mixed symptoms (82), were not assessed.

## Data Availability Statement

The raw data supporting the conclusions of this article will be made available under request to the authors.

## Ethics Statement

The studies involving human participants were reviewed and approved by CEIC EUSKADI. The patients/participants provided their written informed consent to participate in this study.

## Author Contributions

JF-S drafted and wrote the manuscript. SA performed statistical analyses and contributed to data interpretation. IZ, ML, VP, and EV revised the manuscript for important intellectual content and provided scientific advice. IG-O provided funding sources and scientific advice. PS and AG-P designed the study, corrected the manuscript, gave final approval of the version to be published, and ensured the scientific accuracy and integrity of the work. AG-P and PS should be considered joint senior authors. All authors contributed to the article and approved the submitted version.

## Conflict of Interest

VP has been a consultant to or has received honoraria or grants from AB-Biotics, AstraZeneca, Bristol-Myers-Squibb, CIBERSAM, FIS- ISCiii, Janssen Cilag, Lundbeck, Otsuka, Servier, and Pfizer. EV has received grants and served as consultant, advisor, or CME speaker for the following entities: AB-Biotics, Abbott, Allergan, Angelini, AstraZeneca, Bristol-Myers Squibb, Dainippon Sumitomo Pharma, Farmindustria, Ferrer, Forest Research Institute, Galenica, Gedeon Richter, Glaxo-Smith-Kline, Janssen, Lundbeck, Otsuka, Pfizer, Roche, Sage, Sanofi-Aventis, Servier, Shire, Sunovion, Takeda, the Brain and Behaviour Foundation, the Generalitat de Catalunya (PERIS), the Spanish Ministry of Science, Innovation and Universities (CIBERSAM), EU Horizon 2020, and the Stanley Medical Research Institute. AG-P has received grants and served as consultant, advisor, or CME speaker for the following entities: Almirall, AstraZeneca, Bristol-Myers Squibb, Cephalon, Eli Lilly, Glaxo-Smith-Kline, Janssen-Cilag, Ferrer, Johnson & Johnson, Lundbeck, Merck, Otsuka, Pfizer, Sanofi-Aventis, Servier, Shering-Plough, Solvay, the Spanish Ministry of Science and Innovation (CIBERSAM), the Ministry of Science (Carlos III Institute), the Basque Government, the Stanley Medical Research Institute (03-RC-003), and Wyeth. PS has been a consultant to and/or has received honoraria or grants from Adamed, CIBERSAM, European Commission, GlaxoSmithKline, Government of the Principality of Asturias, Instituto de Salud Carlos III, Janssen-Cilag, Lundbeck, Otsuka, Pfizer, Plan Nacional Sobre Drogas, and Servier. The remaining authors declare that the research was conducted in the absence of any commercial or financial relationships that could be construed as a potential conflict of interest.
